# The Road from Host-Defense Peptides to a New Generation of Antimicrobial Drugs

**DOI:** 10.3390/molecules23020311

**Published:** 2018-02-01

**Authors:** Alicia Boto, Jose Manuel Pérez de la Lastra, Concepción C. González

**Affiliations:** Instituto de Productos Naturales y Agrobiología del CSIC, CSIC-Spanish Research Council, Avda. Astrofísico Fco. Sánchez, 3-38206 La Laguna, Tenerife, Spain; jm.perezdelalastra@csic.es (J.M.P.d.l.L.); ccgm@ipna.csic.es (C.C.G.)

**Keywords:** antimicrobial, antibiotic, peptides, AMP, cathelicidins, site-selective, peptide modification, tag-and-modify, customizable units, gene mining

## Abstract

Host-defense peptides, also called antimicrobial peptides (AMPs), whose protective action has been used by animals for millions of years, fulfill many requirements of the pharmaceutical industry, such as: (1) broad spectrum of activity; (2) unlike classic antibiotics, they induce very little resistance; (3) they act synergically with conventional antibiotics; (4) they neutralize endotoxins and are active in animal models. However, it is considered that many natural peptides are not suitable for drug development due to stability and biodisponibility problems, or high production costs. This review describes the efforts to overcome these problems and develop new antimicrobial drugs from these peptides or inspired by them. The discovery process of natural AMPs is discussed, as well as the development of synthetic analogs with improved pharmacological properties. The production of these compounds at acceptable costs, using different chemical and biotechnological methods, is also commented. Once these challenges are overcome, a new generation of versatile, potent and long-lasting antimicrobial drugs is expected.

## 1. Introduction

Host-defense peptides, also called antimicrobial peptides (AMPs), have been used by animals and plants for millions of years as defense against pathogens [1,2,3,4,5,6]. There are several AMP families, such as defensins, dermaseptins, cathelicidins, temporins, plant systemins, and others [2,3,4,5,6]. Although there is a considerable structural variety, in general these compounds are short amphiphilic peptides (generally less than 50 residues), with net cationic charge. Interestingly, some microorganisms produce related antimicrobials composed by hydrophobic and cationic aminoacids [7] (e.g., polymyxins [8], octapeptins [9], gramicidin [10]). These peptides probably allow the producing microorganism to get rid of competitors or to protect their plant or animal hosts. Thus, *Paenibacillus polymyxa* is a nitrogen-fixing bacteria that grows on plant roots; it produces the antibiotic polymyxins that are able to disrupt biofilms of pathogens such as *Pseudomonas aeruginosa* or *Staphylococcus aureus* [11].

AMPs present many advantages as potential antimicrobial drugs: they display a broad spectrum of activity, induce very little resistance, have a synergic effect with conventional antibiotics, and can modulate the immune system and neutralize endotoxins [12,13,14,15,16,17,18,19,20].

They are usually classified in three groups: α-helical, β-sheet, and extended peptides; the latter are usually enriched with certain aminoacids (e.g., proline, lysine). Many AMPs form α-helical structures with cationic and hydrophobic residues oriented on opposite faces of the helix; this facial amphiphilicity is described as “amphipathicity” [21].

Their amphiphilic structure with segregated hydrophobic and cationic regions favours their interaction with anionic bacterial cytoplasmic membranes, followed by their disruption [21,22,23,24,25,26]. According to the “barrel-stave” and “toroidal pore” models, the AMPs form pores in the membrane, while in the “carpet” model, the peptides form micelles with the membrane components, causing membrane destruction. At high concentrations, the peptides can behave as a detergent.

Other less disruptive (non-pore) models have been proposed, such as membrane thinning, depolarization or aggregation. In any case, the mechanism may change according to the peptide concentration, pH, or the temperature [21,22].

The selectivity of AMPs for bacterial membranes over eukaryotic membranes is due to their different composition. Bacterial membranes contain many anionic lipids (cardiolipin, phosphatidylglycerol), in contrast to eukaryotic membranes, which contain zwitterionic lipids (such as sphingomyelin and phosphatidylcholine), and also cholesterol [21]. The cationic peptides are attracted by electrostatic forces to the negatively-charged bacterial membrane, and after adsorption to the cell surface, the peptide hydrophobic residues insert into the membrane (permeation effect) [21,22,23,24,25,26].

Membrane disruption is not the only mechanism of action of these peptides. They can also entangle microbes [27], affect many cytoplasmic processes, such as cell wall, protein and nucleic acid synthesis [1,2,16,19], and hinder the formation of bacterial biofilms [28,29]. In addition, they can act as immunomodulating agents, controlling inflammation and other septic responses to infections [30,31,32]. It has been suggested that many AMPs act on the bacterial invader causing different stresses, until their combined action causes the cell death [16].

In any case, due to their promiscuous mode of action and lack of specificity, AMPs induce little resistance. This fact, together with their broad activity spectrum and synergy with other antimicrobials [12,33], has drawn the attention of the pharmaceutical industry and much work has been carried out to develop AMP drugs [12,13,34,35,36]. 

However, some drawbacks for their clinical use have limited the number of approved AMPs: poor selectivity, hemolytic activity and host toxicity, low stability to protease degradation in vivo, low hydrosolubility and other biodistribution problems, and cost of production [1,12,13]. In addition, although several AMPs have reached advanced clinical trials, most were not approved because they were not superior to the current treatments, as in the case of magainin 2 analogue pexiganan, which was developed for the prevention of diabetic foot ulcers [34,35,36,37]. 

Currently, there are some AMPs approved for clinical use [12,13,34,35,36]. Gramicidins, isolated from *Bacillus brevis*, are used in ophthalmic drops and for the treatment of wound infections and genital ulcers. However, their haemolytic activity prevents their use in systemic infections [10,38,39,40]. Polymyxin B, isolated from *P. polymyxa*, is prescribed to treat eye infections; it is also useful to remove endotoxins, but its nephrotoxic effects limit its applications. Polymyxin E (colistin, and prodrug colomycin) is used to treat infected wounds, but also as last-resort drug against some *Pseudomonas aeruginosa* infections, particularly recurrent ones that affect the lungs in cystic fibrosis [8,41].

The anti-*Candida* drug caspofungin (Cancidas^®^, Merck, USA) is a cyclic hexapeptide with several cationic chains [42]. Caspofungin is a semisynthetic compound derived from fungal echinocandins [43]. However, the natural peptides such as echinocandin B were unsuitable for clinical use, due to their hemolytic activity. To overcome this problem, semisynthetic analogs were developed, but the first drug to enter clinical trials (cilofungin) was discarded due to the toxicity of the vehicle (solvent needed for administration). Finally, caspofungin was approved by FDA for antifungal prophylaxis in stem cell transplant and febrile neutropenia, and in the treatment of refractory aspergillosis and yeast infection. Its toxicity is remarkably low, presents few interactions with other drugs, and as an additional advantage, has a long half-life. However, its oral bioavailability is poor and, therefore, it is administered by intravenous infusion [42,43].

More examples that are in advanced clinical trials (>phase III) are iseganan, derived from pig protegrin, for the treatment of oral mucositis [44,45], and omiganan (ox indolicidin) for the prevention of catheter infections and acne [46,47]. Other AMPs and mimetics are also in clinical trials, such as the defensin mimetic Brilacidin^®^ (Innovation Pharmaceuticals, USA), in Phase II to fight acute skin and soft tissue infections such as oral mucocitis [48,49]. The human defensin Novexatin^®^ (NovaBiotics Ltd., Aberdeen, UK) has been formulated as a brush-on treatment for fungal nail infection, and Phase II studies have shown that is safe and the infection disappears after just one month of daily application [50]. Human LL-37 (also called OP145) is under development (phase II) to treat chronic middle ear infection, and is also studied for manufacturing of biomedical devices [51,52]. The human lactoferrin fragment hLF1-11 is in phase II clinical trials for the treatment of bacterial and fungal infections, and in phase I for prophylaxis in hematopoietic stem cell transplantation [53,54]. PAC-113 (histatin 3) is a 12-amino acid peptide derived from natural histatin found in human saliva; it is used in oral mouth rinse formulation to treat candida infections (Phase II) [55,56,57].

One of the peptides under development (IDR1) is a small synthetic peptide derived from bovine bactenecin [58,59]. Interestingly, the innate defense regulator IDR-1 displayed no antimicrobial properties in vitro, but was highly active in vivo against methicillin-resistant *Staphylococcus aureus* (MRSA) in invasive peritonitis mouse models. This result suggested that IDR-1 presented immunomodulatory properties. In effect, it was observed that the peptide increased the levels of monocyte chemokines, and therefore recruited monocytes and macrophages to fight the bacteria. In addition, it regulated pro-inflammatory cytokine responses to appropriate levels. Other bactenecin derivatives such as IDR-1002 and 1018 have displayed improved properties, and a synergic effect in live infection models [60].

The IDRs highlight the efforts to develop AMPs not only for the treatment of topical infections, but also for systemic ones. Another example in Phase II development is WAP-8294A2 (Lotilibcin), derived from *Lysobacter* sp., for the treatment of systemic infections produced by Gram-(+) bacteria, particularly MRSA and vancomycin-resistant enterococci (VRE) [61]. 

Other antimicrobial peptides in preclinical phase, with promising activities in animal models, will soon enter clinical studies against a variety of fungal and bacterial infections. Some of them are natural peptides or directly derived therefrom (Buforin II, Temporin 10, BacBc) [17,34,35,36], while others are synthetic peptides generated by rational drug design. For instance, Novamycin^®^ (NovaBiotics Ltd., Aberdeen, UK) is a synthetic cationic peptide active against invasive fungal infections caused by *Aspergillus* and *Candida* [62], while Novarifyn^®^ can fight bacterial infections caused by difficult pathogens such as MRSA, *Pseudomonas aeruginosa*, *Clostridium difficile* and *Acinetobacter baumannii* [63]. Although at this early stage it is difficult to predict how many of these new-generation peptides will arrive to the market, their promising activity against pathogens which do not respond to current treatments, together with their improved pharmacological properties, allow us to expect that some AMPs will join the list of approved drugs in the next years [34,35,36].

## 2. Discovery of New AMPs

### 2.1. Biotechnological Discovery Strategies

**Scheme 1:**
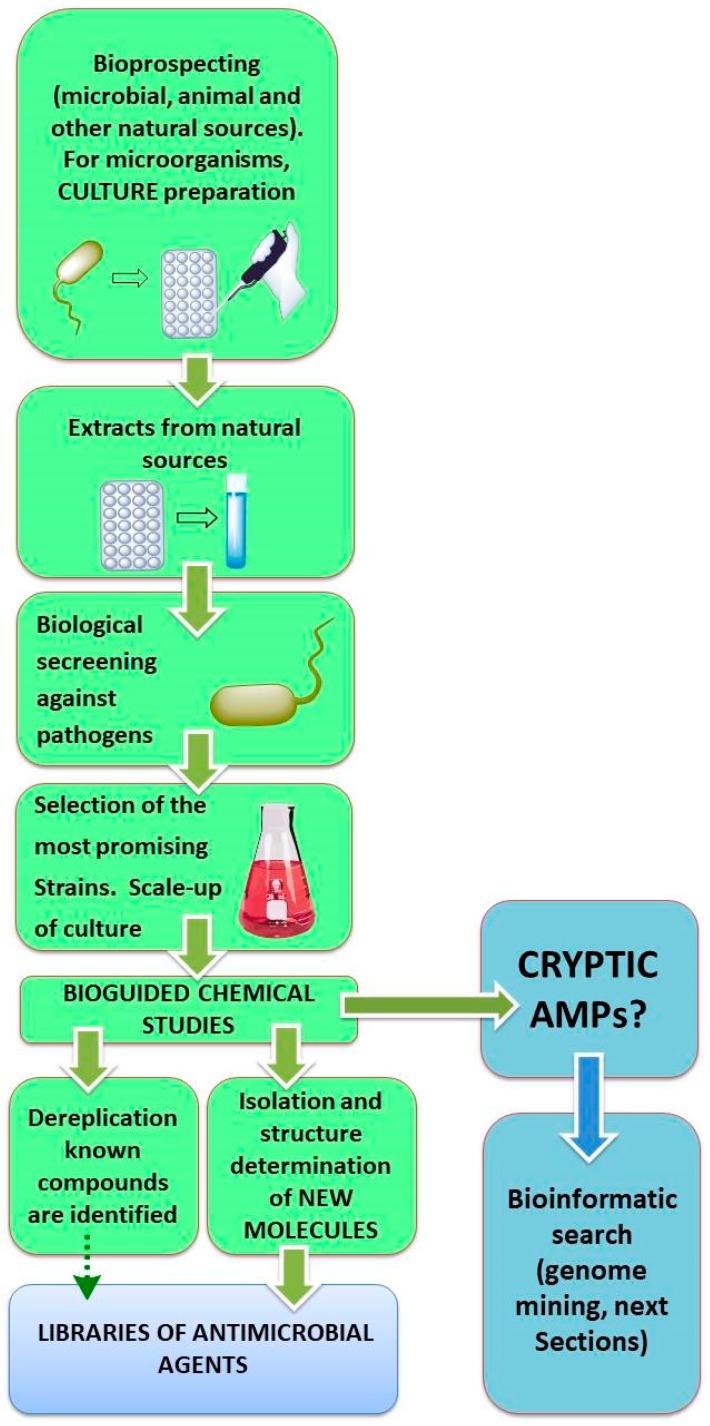
Bioguided search of AMPs.

In the traditional approach to discover new bioactive products, they have been isolated from their natural sources by processing a sample, extraction of the metabolites (e.g., by partition with different solvents), and bioguided characterization of the extracts (Scheme 1, [64]). However, this procedure has limitations. For AMPs, the isolation techniques depend on the amount of peptide available [64,65,66,67,68].

When the peptide is detected in microorganisms, scale-up of the culture and conventional extraction may be possible. However, for the isolation of gene-encoded AMPs, other strategies are preferred. For example, the cDNA encoding the biosynthetic precursor of Dermaseptin-PH was identified from the skin of the south-american frog *Pithecopus hypochondrialis* [65]. The skin secretion underwent initial rapid amplification of complementary DNA (cDNA) ends by PCR (RACE-PCR), which allowed identification of the cDNA encoding the biosynthetic precursor of Dermaseptin-PH. The predicted primary structure was confirmed by identification in the skin secretion by reverse-phase high-performance liquid chromatography (RPHPLC) and MS/MS fragmentation. Then the chemical synthesis of Dermaseptin-PH was carried out, followed by the biological assays. The peptide inhibited the growth of Gram-(+) and Gram-(−)-bacteria, and the fungus *Candida albicans*. 

An important limitation in natural products search is that many interesting products are not expressed by the organism, and their genes remain in a ‘silent’ state until a change in the environment promotes their transcription. For instance, some actynomycetes do not produce antibiotics until they are co-cultured with competing bacteria or until the culture medium is provided with activators such as goadsporin [69].

Clearly, a method to track these ‘cryptic’ or ‘silent’ natural products would be desirable [70,71]. Bioinformatic techniques have been used to predict cryptic products codified in the known genome of different organisms, from microbes to animals. 

**Scheme 2:**
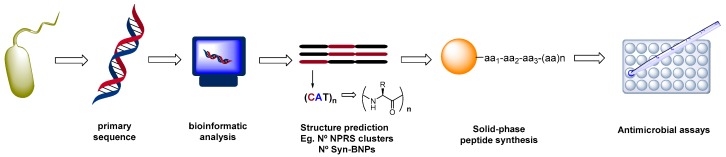
Identification of ‘cryptic’ or ‘silent’ products by bioinformatics techniques.

For instance, Brady et al. have described the discovery of new antibiotics from microorganisms by bioinformatic screening of primary sequences of the biosynthetic gene clusters (Scheme 2, [72]). The structures were then obtained by chemical synthesis to afford synthetic-bioinformatic natural products (syn-BNPs). This approach allowed bypass the culture steps. Brady identified cryptic peptides that could be produced by non-ribosomal peptide synthetases, among them a potent broad-spectrum antibiotic, active against MRSA, and which induced negligible resistance. Another peptide with activity against several fungal pathogens was also discovered, and proved a promising agent for the treatment of vaginal, ear, hand and central nervous system infections.

**Scheme 3:**
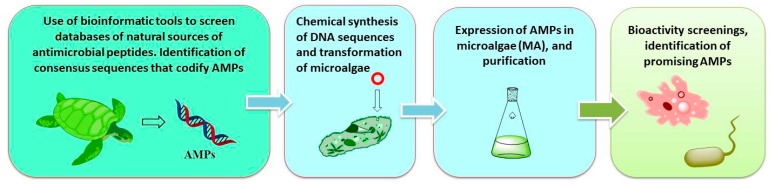
Identification of ‘cryptic’ or ‘silent’ products from animal sources by bioinformatics techniques, and production in microalgae.

It is also possible to carry out this genome mining from other natural sources. Our group has carried out work in the discovery of AMPs (cathelicidins) from animal origin using bioinformatic techniques, and then produced them in transformed microalgae (the eukaryotic *Chlamydomonas reindhartii* and the prokaryotic *Synechococcus elongatus*), as summarized in Scheme 3 [73]. 

Indeed, among the AMPs, cathelicidins are particularly interesting, since they contain highly conserved domains (*N*-terminal cathelin-domain), preserved across evolutionary distant species. Using this domain to search into genome databases, novel cathelicidins were found, from reptiles to bats and birds [74,75,76]. Predicted cathelicidins were identified by screening genome databases [77] using the BLAST tool for DNA sequences [78]. 

The complete sequence of cathelicidins was assembled by joining the exons identified in key contigs using GeneScan [79] and GeneMarK [80] programs. Each DNA sequence thus assembled was translated into amino acids and the resulting protein was subjected to PSI-BLAST to confirm that it was indeed a cathelicidin. The initial protein was processed to release the active peptide, separating it from inactive fragments. 

The AMPs were subjected to in silico analysis of their antimicrobial activity using several bioinformatic tools [21,81,82,83,84,85,86,87]. For example, the online tool APD3 calculates the different parameters related to the possible antimicrobial activity of the peptides (e.g., net charge, length, percentage of hydrophobic residues, helicity) [81,82]. On the other hand, the bioinformatics tool AMPA can predict antimicrobial regions on any protein by assigning an antimicrobial index to each residue [83,84].

In addition, the antimicrobial activity of the peptides was tested against Gram-positive and Gram-negative microorganisms, using the minimal inhibitory concentration assay [88]. The cytotoxicity (hemolytic activity) of the peptides was assesed as well. The most promising peptides were selected for their recombinant production in microorganisms, in order to obtain adequate amounts for other assays. 

The bacteria *E. coli* and yeast were considered first, since their physiology is well-known, there are numerous protocols for their transformation, and vectors that contain a great diversity of promoters and selection markers are readily available [89]. Specific and well-characterized strains can achieve high levels of expression. However, these microorganisms also present disadvantages, such as their potential susceptibility to the harmful antimicrobial peptides. Another drawback would be the need to purify the peptides from the extract, in case that the peptide is trapped as insoluble form in inclusion bodies, and in some cases, the removal of endotoxins. Synthetic biology techniques facilitate the availability of the genetic material that codes for antimicrobial peptides with the codon optimization of the host and it also allows the incorporation of signal peptides, epitopes for the detection of the final product, or the inclusion of appropriate targets for proteolytic enzymes, tags or tails to facilitate purification [90].

An alternative for an expression system of antimicrobial peptides without contaminating endotoxins could be the transformation of eukaryotic cells from animal or human origin. In this case, it may not be necessary to purify or decontaminate the extracts. However, the cost of producing recombinant proteins would be considerably higher than using conventional microorganisms [91].

Several groups have shown that a production system based on microalga could be an alternative to the conventional fermentation systems that use bacteria, yeast, or mammalian cells [73,92,93,94,95]. First, the antimicrobial peptide is less likely to be toxic to this production system. Besides, microalgae allow greater scaling-up volumes, spread over large areas, making these production systems very profitable. In spite of smaller production yields, microalgae offer a higher benefit/cost ratio compared to conventional systems. Another asset is that some microalgae are considered GRAS (Generally Regarded as Safe), and in these cases and for certain commercial uses, purification processes may not be necessary. For example, applications for cosmetics, topical medicines, pesticides for agriculture, food conservation, etc. would not require peptide purification.

There are already numerous tools for the efficient transformation microalgae. For example, in the case of the microalga *Chlamydomonas reindhartii*, it is possible to transform the three genomes: nuclear, chloroplastic and mitochondrial. Once transformed, microalgae can be grown in photobioreactors, in controlled conditions and free of pathogens. On the other hand, controlled conditions would avoid release of the transformed microorganism into the environment, and would improve the biosecurity of the final product [92].

**Figure 1:**
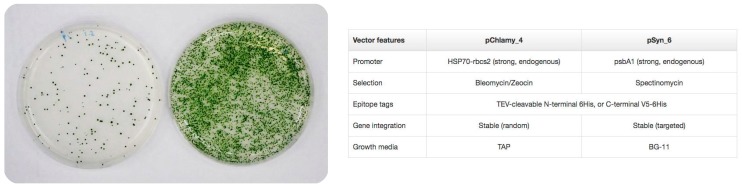
(**Left**) Transformed *S. elongatus* growing in BG-11 agar plates containing spectinomycin (5 µg/mL). Negative control with no insert (**Left**) and containing the bird cathelicidin peptide Mm_KR-26; (**Right**) Comparison of the microalga vectors pChlamy_4 and pSyn_6 (ThermoFisher) used for the recombinant expression of cathelicidin antimicrobial peptides into the microalga *C. reindhartii* and *S. elontgatus*, respectively.

Perez de Lastra et al. have used the eukaryotic microalgae *Chlamydomonas reindhartii* and the prokaryotic *Synechococcus elongatus* as expression systems. Both the prokaryotic and the eukaryotic microalga have strong, endogenous promotors and can introduce 6xHis Tags for the purification of the recombinant proteins (Figure 1). Using the commercially available kits for microalga expression, the two microalgae were transformed for the recombinant expression of several cathelicidins. Some of the isolated cathelicidins were particularly effective against both Gram-(+) and Gram-(−) bacteria, and besides, displayed low haemolytic activity in human erythrocytes [73]. These results highlight their potential for use in different formulations and/or pharmaceutical, cosmetic and/or phytosanitary compositions.

### 2.2. Chemical Synthesis in Discovery Strategies

Antimicrobial peptides are usually isolated in very small amounts from the natural sources. Therefore, a chemical synthesis is often implemented to obtain adequate amounts for proper peptide characterization and for the biological assays [96,97,98,99]. Moreover, the chemical synthesis can provide analogues of natural peptides with improved properties, such as superior potency, selectivity, stability or biodistribution, and is a key technology in drug discovery and development programs [1,16,100]. The following sections deal with the advances in synthetic methodologies to provide suitable amounts of AMPs, and also to diversify peptide libraries. Then, the use of libraries for the determination of structure-activity relationships (SAR) will be discussed, and how this knowledge has led to the development of semi-synthetic or synthetic analogues with better pharmacological profiles.

#### 2.2.1. Advances in Synthetic Methodologies: Providing Scarce AMPs and Selected Analogues

When an antimicrobial peptide is scarce in nature, chemical synthesis can provide in a relatively short time suitable amounts for its complete characterization and for biological screening [98,99]. In addition, the synthesis of selected analogues is also possible.

**Scheme 4:**
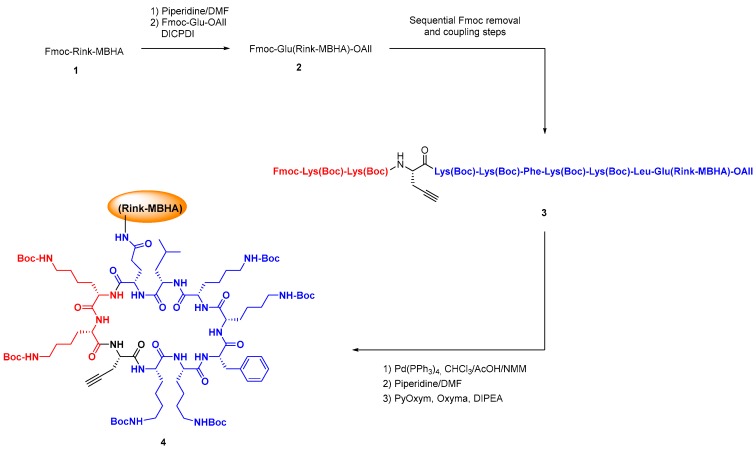
SPPS in the preparation of peptides with non-proteinogenic units, and cyclization of a BPC precursor.

The standard solution and solid phase synthesis of small and medium-size peptides is well-known [96,97,98,99,100], but the preparation of difficult sequences can pose many problems, and has been reviewed by Albericio et al. [101]. In the case of cyclic peptides, the cyclization may be troublesome, and different approaches have been developed, from dilution conditions to the development of new coupling reagents, use of enzymes, use of native peptide ligation methodologies, etc. [102,103,104,105]. Scheme 4 shows the solid-phase synthesis and cyclization of an antimicrobial BPC precursor (conversion **1**→**4** [106]). 

Other synthetic challenge is the production of disulfide bonds, which is usually carried out at the end of the synthesis, as will be shown later for the synthesis of snakins [107] and θ-defensins [108]. As a result, peptide analogues with disulfide bond surrogates (such as C-C or C-S bonds) have been developed [109].

**Scheme 5:**
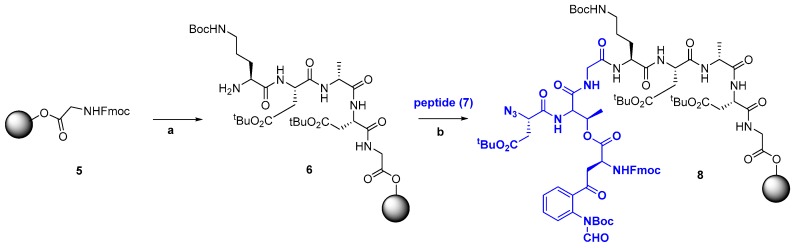
Ligation of peptides in the synthesis of a precursor of polymyxin E. *Reaction conditions*: (**a**) Fmoc-SPPS; (**b**) HATU, DIEA, DMF, 45 min.

**Scheme 6:**
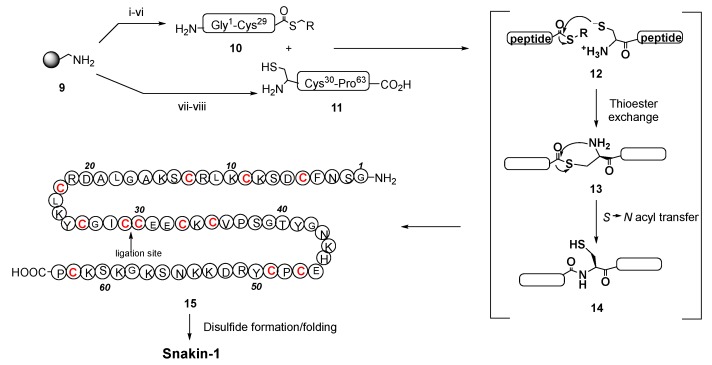
Synthesis of a precursor of snaking-1. *Reaction conditions*: (i) Boc-Ala-Pam, DIC, CH_2_Cl_2_, 1 h; (ii) TFA; (iii) TrtSCH_2_CH_2_CO_2_H, HBTU, DIPEA, DMF, 16 min; (iv) TFA, TIPS, H_2_O; (v) Boc-Cys-(4-MeBn)-OH, HBTU, DIPEA, DMF, 1 h; (vi) Boc SSP; (vii) Boc-Pro-Pam, DIC, CH_2_Cl_2_, 1 h; (viii) Boc SSPS. TIPS = triisopropylsilane; DIC = diisopropylcarbodiimide; DIPEA = diisopropylethylamine; DMF = *N,N*-dimethylformamide; HBTU = *O*-benzotriazole-*N,N,N*′,*N*′-tetramethyluronium hexafluorophosphate; Pam = phenylacetamidomethyl; Trt = triphenylmethyl.

For the generation of large peptides, several small or medium-size fragments are synthesized and then attached. Usual ligation methods are solution or solid-phase fragment condensation (Scheme 5), and native chemical ligation (Scheme 6). In Scheme 5, the pentapeptide **6** is produced by solid-phase synthesis, and then coupled to fragment **7** to give a precursor **8** of a polymyxin analogue [110]. In the final analogue, the Dab-3 residue was replaced by a Dap-3 unit, and a synthetic, more polar side-chain was introduced. The analogue was active in vitro against Gram-(−) bacteria, such as *Acinetobacter baumanii* and *Pseudomonas aeruginosa* [8,41,110].

For the synthesis of snakin-1, an antimicrobial peptide produced by several plants, such as potato, rice, maize, soybean, and strawberry, a native chemical ligation method was used. As shown in Scheme 6, two fragments **10** and **11** were produced by solid-phase synthesis, and then ligated (conversion **12**→**14**) to give the snakin precursor **15**. Oxidation of the cysteine units generated six internal disulfide bonds, providing the folded snakin-1 in about 50% yield [107]. The native chemical ligation has also been used to cyclize peptides, as in the case of θ-defensins [108].

The advances in the chemical synthesis have also affected the generation of peptide libraries for SAR studies. Thus, in the traditional way to prepare peptide libraries, each library member is prepared de novo. The introduction of a new amino acid involves two steps, namely the deprotection of the terminal unit and the coupling of the new residue. When large peptides are synthesized, or when difficult sequences or macrocycles need to be prepared, the conventional process can pose problems and be costly in time and materials. In addition, since for many activity studies only a small part of the peptide needs modification, an alternative strategy would be preferrable, where a few starting peptides undergo a site-selective modification of certain positions, which does not affect to the other positions [111].

**Scheme 7:**
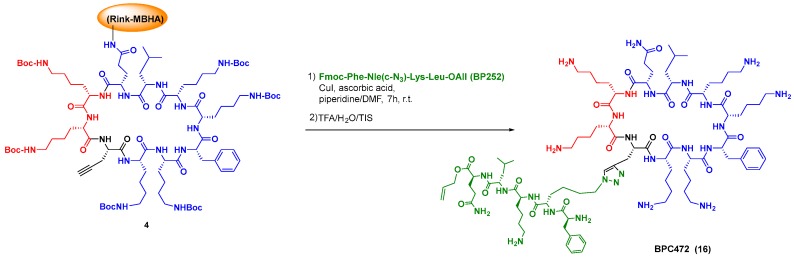
Peptide ligation using click’ chemistry.

Although this strategy represents a synthetic challenge, due to the similar reactivity of the amino acid units, in the last years a variety of methods have allowed the selective modification of peptides and proteins. In the tag-and-modify approach, a functional group in a residue (tag) is replaced or transformed into other ones. For instance, amino acids with haloaryl units undergo sp2-sp2 couplings [112]. Click chemistry provides a practical method for the introduction of different groups and even the ligation of peptide fragments, as shown by the synthesis by Planas et al. of cyclic peptidotriazoles derived from the antimicrobial peptide BPC194 (Scheme 7, conversion **4**→**16**, [113]). Some of them presented potent activity against plant pathogens, from bacteria to fungi, while producing low hemolysis, and were quite stable towards protease cleavage [106,113,114].

**Scheme 8:**
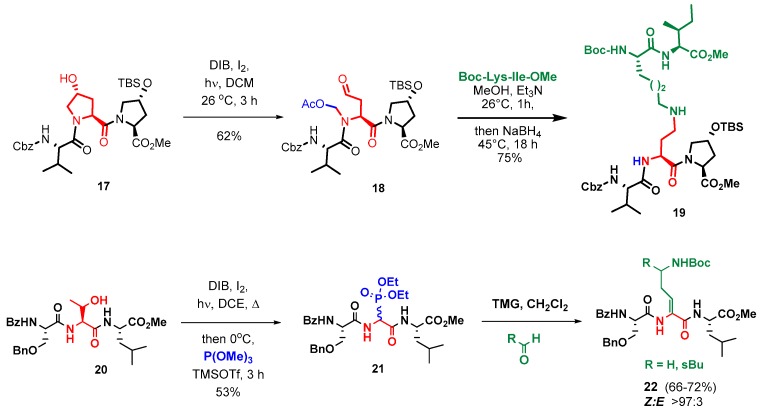
Site-selective modification of peptides in the discovery of new AMPs.

In a second approach for the generation of peptide libraries by site-selective modification, the core of the starting amino acid (customizable unit) undergoes a scission process and is converted into a very different one (e.g., by cleavage of the lateral chain and attachment of a new one). Recently, our group has introduced new customizable units that allow the site-selective modification of peptides and the creation of both cationic or hydrophobic residues [111,115,116,117,118,119]. Peptide ligation is also possible, as shown in the Scheme 8. This strategy has allowed the synthesis of peptide libraries with arylglycine, 4-aminohomoalanines, dehydroamino acid units, β- and γ-amino acid residues, and many other residues. Some of these compounds have displayed a promising activity against plant and animal pathogens.

#### 2.2.2. AMP Libraries for SAR Studies

**Figure 2:**
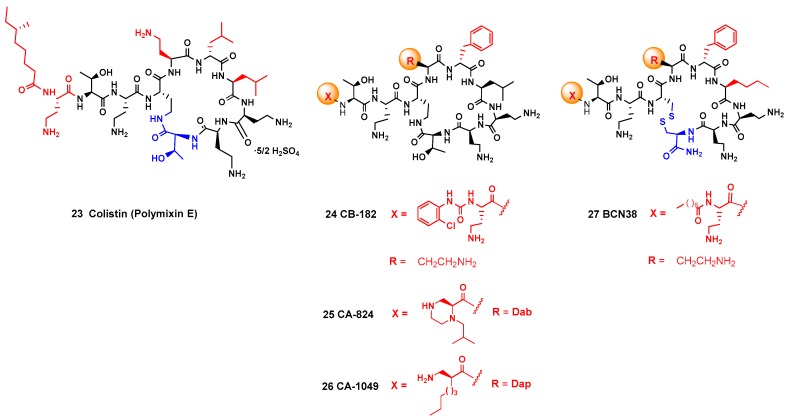
Structures of colistin A, its analog CB-182,804, the Cantab analogs CA-824 and CA-1049, and the Barcelona disulfide analogs. The positions were changes were made are displayed in colors.

Once a promising antimicrobial peptide has been identified, SAR studies are carried out to determine the structural features which are key for the activity. Since a polymyxin derivative, colistin, is one of the AMPs in clinical use, different analogues have been produced (Figure 2, [8,41,110]). In the Pfizer 5x, MicuRx and CB-182,804 analogs, the fatty acid group in the lateral chain is replaced by other acyl or carbamate protecting groups. In the Monash FADDI-, Queensland-, Cantab-, and Northern Antibiotic analogs, the terminal acyl group and the amino acids in the cyclic core are changed. In addition, in the Barcelona analogs, a disulfide group is introduced in the cyclic core. Some of these analogs displayed a potent antibacterial activity. A similar study was carried out for the structurally related octapeptins [9]. In related studies for other family of antimicrobial peptides, Inoue has carried out the synthesis of lysocin E, preparing enantiomeric, epimeric, and *N*-demethylated analogues, and also different amino acid residues [120]. In this way, structure-activity (SAR) relationships have been determined, such as the influence of cationic, hydrophobic and aromatic units on the antibacterial activity [121].

These studies have shown that the ratio between cationic and hydrophobic units is essential for the antimicrobial activity but also for the selectivity. Thus, Fattorusso has reported a new analogue of temporins [122], the defense peptides isolated from frogs (*Rana temporaria*). Temporins are small peptides (8–14 residues) with amides at the *C*-terminal position, and a small positive charge at neutral pH. The analogue presented one point mutation (G6A) in the temporine sequence and two extra lysines at the *N*-terminal position. This peptide was active against Gram-positive and Gram-negative bacteria, and displayed no hemolytic activity. On interaction with bacterial membranes, the unnatural peptide used one Lys residue to anchor the peptide to the bacterial membrane, while the other Lys unit allowed penetration of the micellar structure. 

This understanding has prompted the development of peptide analogues and peptidomimetics where different ratios of hydrophobic and cationic residues were tested. With the right balance, truncated sections of AMPs and even very small peptides can display antimicrobial activity [28,29,123,124,125,126,127,128,129,130].

Thus, structural simplification by truncation has been used with human cathelicidin LL-37 [123], generating several fragments which outperformed the antibiofilm effect of LL-37 against MRSA strains. Besides, truncation of LL-37 decreases its cytotoxic effects while the antimicrobial activity is maintained. Montesinos and Bardají have reviewed the shortening of linear AMPs [124], including magainin (pexiganan, MSI-99, ESFs), protegrins (iseganan), cecropins (D4E1) and tachyplesins (TPY).

A similar effect was observed when linear analogues of the cyclic pepide battacin were produced. Conjugation of a linear analog with a smaller fatty acid, 4-methylhexanoic acid, gave a lipopeptide with superior antibacterial activity and selectivity. In addition, antibiofilm activity against *S. aureus*, *P. aeruginosa* and *P. syringae* was observed [28]. 

Plant systemins served as inspiration for synthetic small-size peptides such as FRLKFHF that have been developed as pesticides in agriculture [124]. In another example, natural sapecin B inspired a short synthetic peptide BF2 (RWRLLLLKKH) with a potent activity against vancomycin-resistant enterococci at low micromolar range, also displaying activity in vivo. The peptide was non-toxic to mammalian cells [125]. 

**Figure 3:**
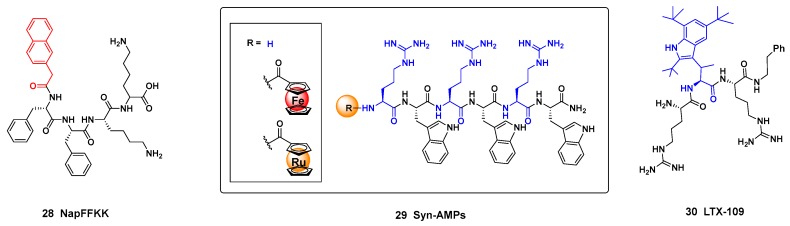
Structures of AMP analogues: (**Left**) the NApFFKK peptide; (**Center**) Synthetic AMPs with ferrocene or rutacene groups; (**Right**) Unnatural tryptophan units in a ferritin-inspired peptide, LTX-109.

An even shorter peptide NapFFKK **28** (Figure 3, on the left), with a terminal naphthalene ring, displayed a potent antimicrobial activity [130]. The peptide self-assembled and its hydrogels greatly reduced the formation of *S. epidermidis* biofilms. Interestingly, it was observed that reducing the α-chain lenght at the cationic residues, the biofilm activity was reduced. The authors suggested that with shorter chains, the amine group did not interact well with the negatively charged bacterial membranes. A good activity/citotoxicity ratio was observed.

Some synthetic antimicrobial peptides alternate residues with aromatic and with cationic chains. In a recent work, the structure-activity relationships of the synthetic AMPs **29** (Figure 3, center) was explored using a ferrocene scan [131].

The introduction of unnatural hydrophobic and cationic residues into the peptides can improve the antimicrobial activity and the in vivo stability [132]. After systemic truncation and optimization of peptides derived from lactoferricin, novel synthetic antimicrobial peptidomimetics (SAMPs) were identified as drug candidates, and one of them (LTX-109 (**30**), Figure 3, right), is in clinical trials against bacterial topical infections (Lytix Biopharma AS) [133]. In addition, LTX-109 displayed high antifungal activity against candidiasis and onychomycosis, superior to the current reference drugs, and high selectivity [134].

The stereochemistry of the residues can also influence the antimicrobial activity. The introduction of residues with d-amino acids can increase the proteolytic stability. The replacement of l- by d-amino acids in human cathelicidin LL-37 decreased degradation by proteases and increased the antimicrobial activity [135]. Besides, this replacement can increase drug selectivity; thus, peptides composed solely of l-units have usually high helicity and propensity to form pores both in bacterial and eukaryotic membranes. When some of the l-units are replaced by d-residues, the helicity decreases and the peptides selectively interact with the bacterial membranes, causing less hemolysis while maintaining the antibacterial activity [136].

**Figure 4:**
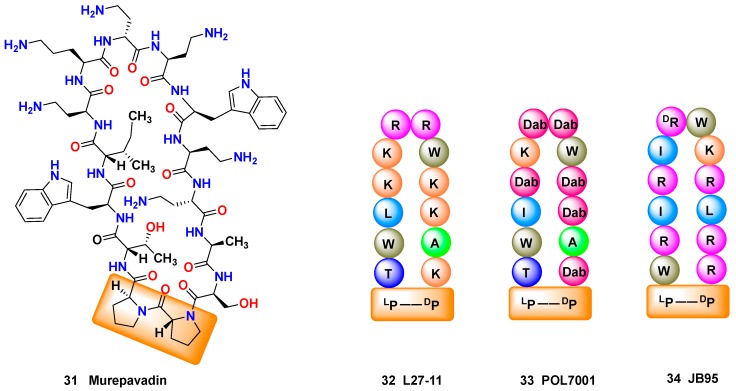
(**Left**) Murepavadin (**31**), an anti-pseudomonas peptide in Phase II trials, with a l-Pro-d-Pro unit that induces a β-turn in the β-hairpin structure; (**Center**) The 12-residue β-hairpin mimetic L27-11 (**32**) with a potent and specific antipseudomonas activity; optimization of this hit generated POL7001 (**33**) (Dab = L-2,4-diaminobutyric acid); (**Right**) Another synthetic peptidomimetic antibiotic JB-95 (**34**), active against *E. coli*.

The introduction of d-amino acids can also favor the formation of secondary structures such as β-turns. The development of β-hairpin epitome mimetics has been reviewed by Robinson [137]; in Figure 4, a l-Pro-d-Pro unit is vital for the formation of a β-hairpin mimetic scaffold incorporating cationic and hydrophobic units. The l-Pro-d-Pro unit can be found in murepavadin **31** (Figure 4, left), which has recently completed phase-II clinical trials for the treatment of life-threatening lung infections caused by *Pseudomonas sp*., and is starting Phase III studies. Interestingly, murepavadin was developed by optimization of a hit, compound L27-11 (**32**), which displayed a potent and selective anti-pseudomonas activity [138]. The mechanism of action of L27-11 was new, since the peptide selectively interacted with the β-barrel protein LptD in Pseudomonas spp., which is involved in lipopolysaccaride (LPS) transport to the membrane [139]. However, L27-11 was not suitable as a drug, since it was rapidly degraded by proteases in human serum. Structural optimization led to compound **33** (POL7001), where many of the arginine (R) or lysine (K) residues were replaced by Dab units (diaminobutyric acid), resulting in greater stability against proteases. Further optimization provided murepavadin **31**. The research on other analogues provided a different β-hairpin peptidomimetic JB-95 (**34**) which was active against *Escherichia coli* [140].

**Figure 5:**
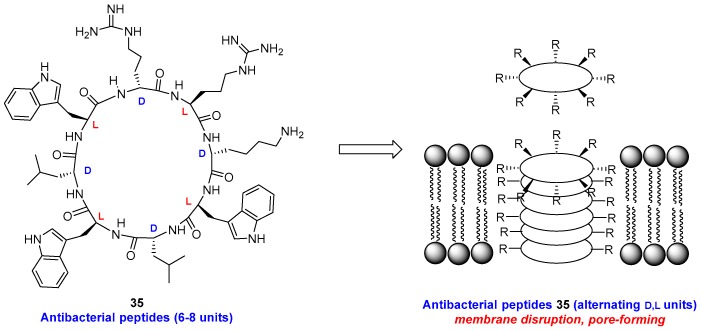
Cyclic peptides with alternating d, l-units self-assemble and form pores in the bacterial membrane.

Alternating d- and l-units in 6–8-membered cyclic peptides **35** (Figure 5) induce the formation of tubular structures which disrupt the bacterial membrane [141,142]. The combination of l- and d-residues generate flat rings with the lateral chains oriented on the outside, which can form intermolecular hydrogen bonds very efficiently. The presence of two or three basic chains gave derivatives with micromolar activity against MRSA, while acid groups reduced the activity due to electrostatic repulsion of the negatively charged bacterial membrane. 

Cyclic peptides, as those in the previous example, are more resistant than linear peptides to proteolytic cleavage. Thus, the cyclic peptide BPC194 [cyclo(KKLKKFKKLQ)] hardly shows degradation by proteinase K after 45 min, while the linear analog BP100 (KKLFKKILKYL-NH_2_) underwent 44% degradation in that time [124]. The ring size [143] and the flexibility [144] of the ring backbone also affect the bioactivity.

In summary, the generation of peptide libraries and the subsequent SAR studies have shed light on the structural features required for antimicrobial activity. As a result, completely synthetic AMPs (SAMPs) and then peptidomimetics have been developed in an effort to improve selectivity, stability, bioavailability and also to reduce costs. The generation of SAMPs has been commented before. In the next section, the development of peptidomimetics will be discussed.

#### 2.2.3. SAR Studies: Natural AMPs as Inspiration for Peptidomimetics

In a further stage, chemical synthesis has allowed the preparation of peptide analogues where at least part of the α-amino acid units have been replaced by other structural elements. However, the principle that hydrophobic and cationic structures must be balanced is still applied. 

**Figure 6:**
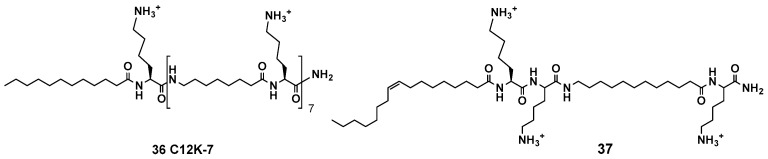
AMP analogs; acyllysine oligomers (OAKs) with alternating acyl (A) and lysine (K) units.

For instance, Mor et al. have designed acyllysine oligomers (OAKs) with alternating acyl chain (A) and cationic lysine (K) units (such as compounds **36** and **37**, Figure 6) [145]. The design intended to avoid the formation of stable secondary structures, while allowing the generation of the active structures on interaction with cell membranes [146]. The studies on the variation of the A chain lenght, and the number of A and K units, finally afforded peptidomimetics with an optimal balance of the overall charge and hydrophobicity. Some of these OAKs have a potent activity against many bacterial strains at low micromolar range. Interestingly, the mechanism of action is variable, from cell membrane disruption to inhibition of DNA replication, according to environmental factors such as pH, temperature and salt concentration [147]. 

**Scheme 9:**
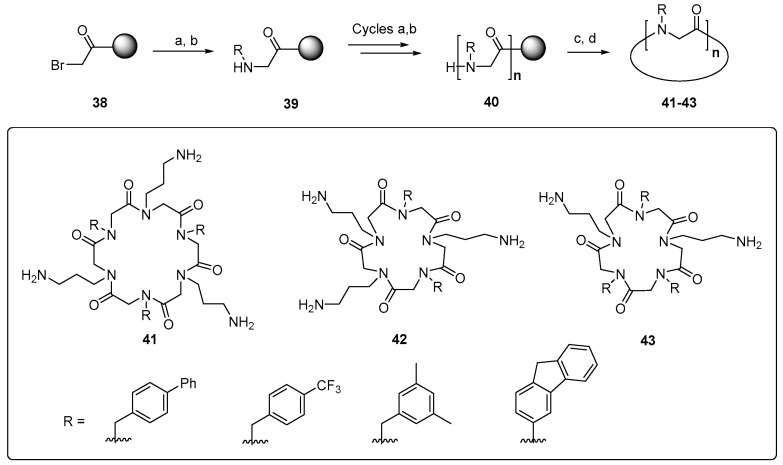
Peptoid analogs, alternating cationic and hydrophobic chains. Cycle **41** gave the best results. *Reaction conditions* (above): (**a**) R-NH_2_, base; (**b**) BrCH_2_COOH; (**c**) 20% HFIP, CH_2_Cl_2_; (**d**) PyBOP, DIEA, DMF. Conversion yields >95% were observed in the synthesis of cyclic peptoid analogues.

Other useful peptidomimetics are peptoids, glycine oligomers where the “lateral chains” are attached to the amide nitrogen. The amphiphilic cyclic peptoids **41**–**43** shown in Scheme 9 present cationic chains, resembling the ornithine units, and hydrophobic chains containing aromatic groups. 

**Figure 7:**
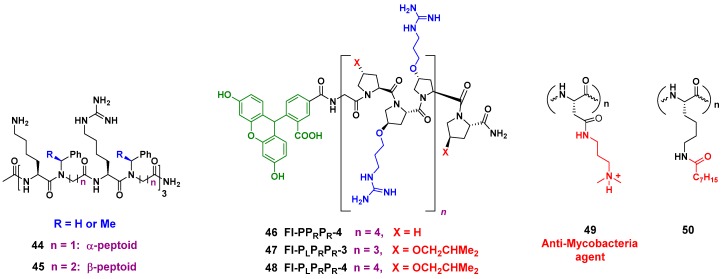
AMP analogs: (**Left**) α- and β-peptoids **44** and **45**; (**Center**) substituted PrAMPs **46**–**48**; (**Right**) introduction of cationic chains in analogues **49** and capped-termini derivatives **50**.

These peptidomimetics are readily synthesized on a solid support through cycles of bromoacetylation and substitution of the halogen group by primary amines. The peptoids disrupt *S. aureus* membrane through the formation of pores, and display a potent activity against methicillin-resistant *S. aureus* (MRSA). A good selectivity is achieved, and as an additional advantage, the unnatural backbone is more resistant to proteases [148]. A related strategy is carried out with the α-peptide/peptoid hybrids **44** and **45** (Figure 7, left), where α-aminoacids with cationic chains alternate with *N*-benzyl glycines. By introducing changes in the lateral chains and the *N*-substituents, a decreased cytotoxicity and a high activity against multidrug-resistant *E. coli* are obtained [149]. Other interesting peptidomimetics are lipidated cyclic γ-AApeptides, where the amino groups in the macrocyclic ring are attached to cationic chains. These compounds disrupt bacterial membranes but also induce the immune response and display an anti-inflammatory effect [150].

Unnatural Proline-Rich Peptides were also used to introduce cationic and hydrophobic “lateral chains” on the backbone, but in the case of compounds **46**–**48** (Figure 7, center), the substituents were attached to the 4-hydroxy group of the Hyp (hydroxyproline) units. It must be said that Proline-rich antimicrobial peptides (PrAMPs) represent promising agents against Gram-negative bacteria, and these new derivatives are active both against Gram-positive and Gram-negative bacteria. Library screening identified a derivative with antimicrobial activity in the low micromolar range but low cytotoxicity, which was superior to the reference compound mellitin [151].

Finally, two interesting (and opposite) strategies to introduce cationic and hydrophobic units are shown in Figure 7 (right, compounds **49** and **50**). In the first example, cell-penetrating synthetic antimicrobial peptides (SAMPs) are prepared using an aspartic acid oligomer, which is amidated, transforming the anionic aspartic unit into a cationic residue. The compounds **49** display potent and selective bactericidal effect against Mycobacterium (*M. smegmatis*), by interacting with its DNA [152].

The opposite approach, “capping” lysine residues, decreases the cationic nature of the peptide. Thus, the activity of short synthetic antimicrobial peptides was modulated by lipidation of the *C*- or *N*-terminal lysine units (compound **50**). Lipidation greatly increased the activity against Gram-positive and Gram-negative bacteria, and a derivative was isolated with activity against *S. aureus*, *A. baumannii* and *P. aeruginosa* in the low micromolar range [153].

**Figure 8:**
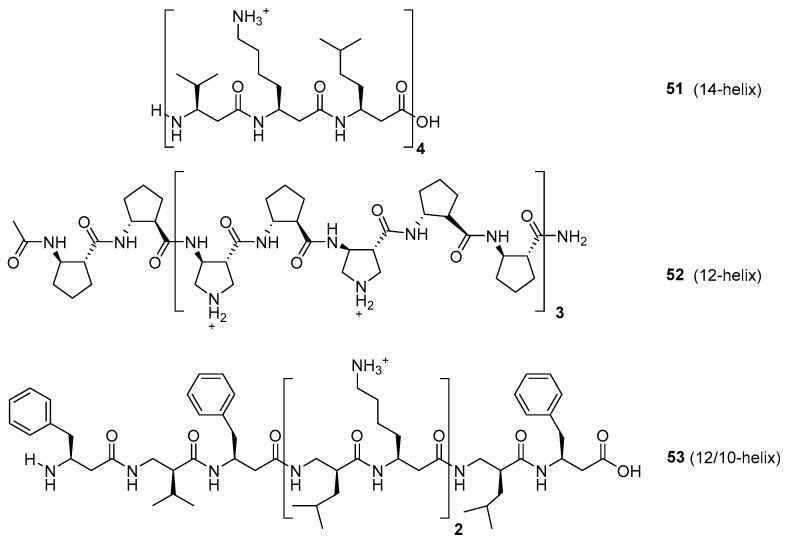
β-peptides with alternating hydrophobic and cationic units and different folding patterns.

The development of AMPs with aminoacid analogs, such as β-amino acids, has also received much attention. Thus, α,β-peptide hybrids and β-peptides often achieve well-defined conformations, and are more resistant to protease degradation. β-Peptides (such as compounds **51**–**53**) usually form globally amphiphilic helices (Figure 8); the groups of Gellmann, Seebach, De Grado, Fülöp, and others have made seminal contributions to understand their folding patterns and their relationships with biological activity [154,155,156,157,158,159,160,161]. Although in the initial studies the β-peptides displayed undesirable haemolytic properties, a better understanding of these foldamers has resulted in the development of β-AMPs with potent antimicrobial activity and low cytotoxicity (hemolysis).

Other amino acid analogues incorporate heteroatom functions, such as the silaamino acids [162]. Incorporation of silicon-containing amino acids in peptides increases their lipophilicity and their proteolytic stability. Thus, when the antimicrobial peptide alamethicin was replaced by a derivative containing β-silicon-β3-amino acids, a 20-fold increase in membrane permeabilization was observed. In another example, helical sulfono-γ-AApeptide foldamers were described, which were resistant to proteolytic degradation. The hit compound presented potent activity against multi-drug-resistant Gram-positive and Gram-negative pathogens [163]. The synthesis of other peptidomimetics, with structures less related to peptides, but which mimic the action of membrane active proteins, has been recently reviewed [164].

## 3. Delivery and Bioavailability Issues

Although beyond the scope of this review, it is worth mentioning that the stability and bioavailability of AMPs can be enhanced by loading them into different delivery systems, such as liposomes, lipic discs, polymer nanoparticles, inorganic nanoparticles, graphene and carbon nanotubes, quantum dots, etc. These systems have been recently reviewed by Piotrowska et al. [165].

## 4. Scale-Up: Overcoming Production Cost Problems

The cost of production can be a limiting factor for the development of AMP drugs. However, biotechnological or chemical production of AMPs and their mimetics have undergone many advances, decreasing the synthetic costs and making large-scale production feasible.

The methodologies that allowed the efficient generation of peptide libraries during the discovery process, are also useful for the industrial production of small peptides (4–10 residues) [166]. Thus, several lipopeptides and other small peptides (<6 units) have been prepared by solution synthesis or by chemoenzymatic methods [167]. The ongoing progress in the field has allowed the synthesis in a multi-tonne scale of the antiviral agent enfuvirtide (Fuzeon^®^, Genentech, USA), a 36-residue peptide [168]. The economical large-scale production of peptide mimics with simpler structures is also feasible, as commented before. However, the chemical synthesis of large peptides at reasonable costs is still challenging. A biotechnological approach is then preferred.

Many large peptides have been produced in microorganisms [169]. However, the generation of AMPs has specific problems, since these peptides can be toxic for the producer cell and are also prone to proteolytic degradation. Therefore, several strategies have been developed, such as the use of fusion proteins, tandem polypeptides, secretion as inclusion bodies, etc. The large-scale production of plectasin, a fungal defensin from *P. nigrella*, has been achieved in high yield and purity using recombinant techniques with the fungus *Aspergillus oryzae* [170]. Being an eukaryotic organism, *Aspergillus* is less sensitive to the antimicrobial peptide. However, for the expression in the bacteria *Escherichia coli* of several AMPs, Vogel used calmodulin (CaM) as a carrier protein [171]. CaM presents a structure with two binding domains which can accommodate different chains containing basic and hydrophobic units, avoiding the toxic effects of the amphipathic AMPs and at the same time, protecting them from degradation during the production process. With this technique, several AMPs were produced, such as melittin, fowlicidin-1, tritrpticin, indolicidin, puroindolide A peptide, magainin II F5W, lactoferrampin B, MIP3α51−70, and human β-defensin 3 (HBD-3). These peptides, in the absence of CaM, would be very toxic to *E. coli* and would kill the producer cells. 

In another example, Kalman described the expression in *E. coli* and the purification of seven recombinant AMPs, in industrial scale [172]. Fusions to sumoase protease (SUMO) produced high yields of the intact recombinant AMPs without toxicity to the bacteria; in addition, a cost-efficient, two-step method for the purification of the recombinant peptides was developed. The peptides IDR1, MX226, LL37, CRAMP, HHC-10, E5 and E6 were produced using this methodology, under the GMP conditions required for human-use drugs. On the other hand, Wink achieved recombinant production of the folded, disulfide-containing Snakin-2 in *E. coli* [173]. The use of microalgae to express recombinant peptides has been commented in Section 2.1. Other examples [124,174] and the promising use of plants as biofactories to obtain crop-protecting peptides has been recently reviewed by Montesinos and Bardají [124]. For instance, in a recent work from the group, the peptide cecropin A was expressed in rice seeds [175,176].

Some years ago, the biotechnological processes were limited to AMPs containing proteinogenic amino acids that could be synthesized in the bacterial ribosomes. Recently, different methods have been introduced to ‘reprogram the genetic code’ and allow the assembly of nonproteinogenic amino acids in the ribosomes. These methods have been recently reviewed by Suga [177], and include in vitro translation, engineered aminoacyl tRNA synthetases, and RNA ‘flexizymes’, offering extraordinary opportunities for the preparation of ‘tailored’ peptides with improved properties.

## 5. Perspectives and Conclusions

Antimicrobial peptides (AMPs), also called host-defense peptides for their immunomodulatory properties, could be the next-generation of antimicrobial “superdrugs” if the problems related to their stability, bioavailability, and production at reasonable costs are solved. Their potent activity against pathogens which do not respond to current treatments, their multiple mechanism of action which results in very low or negligible induction of resistance, their synergy with other antimicrobials, and their ability to modulate our immune response are clear assets for the pharmaceutical companies.

However, currently only a few antimicrobial peptides have reached the market, and most of the approved drugs are for topical use. But in recent years, a better understanding of their physical features and their complex mechanism of action has allowed the development of analogs and mimetics with improved pharmacological properties. On the other hand, the development of new biotechnological and chemical processes for their industrial production has considerably reduced their costs. Currently, there are promising products in pharmaceutical pipelines with excellent results in animal models or in early clinic trials. Interestingly, some of them are intended to fight systemic infections produced by the top-ten pathogen threats. Although not all of these drug candidates will reach the market, it is reasonable to expect that some AMPs will join the list of approved drugs in the coming years. If these expectations are met, a new generation of versatile, potent and long-lasting antimicrobial drugs will soon be available.
